# The clinicopathologic and molecular features, and treatment outcome of fumarate hydratase-deficient renal cell carcinoma: a retrospective comparison with type 2 papillary renal cell carcinoma

**DOI:** 10.18632/aging.205549

**Published:** 2024-02-19

**Authors:** Junjie Bai, Xiaoyan Li, Yahui Wen, Qing Lu, Ru Chen, Rong Liu, Tong Shangguan, Yushi Ye, Jun Lin, Weizhong Cai, Deyong Kang, Jianhui Chen

**Affiliations:** 1 Department of Urology, Fujian Medical University Union Hospital, Fuzhou 350001, Fujian, P.R. China; 2 The Graduate School of Fujian Medical University, Fuzhou 350000, Fujian, P.R. China; 3 Department of Pathology, Fujian Medical University Union Hospital, Fuzhou 350001, Fujian, P.R. China; 4 Department of Breast Surgery, Fujian Medical University Union Hospital, Fuzhou 350001, Fujian, P.R. China; 5 Department of General Surgery, Fujian Medical University Union Hospital, Fuzhou 350001, Fujian, P.R. China

**Keywords:** renal cell carcinoma, hereditary leiomyomatosis and renal cell carcinoma, fumarate hydratase, papillary, immunohistochemistry, molecular

## Abstract

Background: To compare clinicopathologic, molecular features, and treatment outcome between fumarate hydratase-deficient renal cell carcinoma (FH-dRCC) and type 2 papillary renal cell carcinoma (T2 pRCC).

Methods: Data of T2 pRCC patients and FH-dRCC patients with additional next-generation sequencing information were retrospectively analyzed. The cancer-specific survival (CSS) and disease-free survival (DFS) were primary endpoint.

Results: A combination of FH and 2-succino-cysteine (2-SC) increased the rate of negative predictive value of FH-dRCC. Compared with T2 pRCC cases, FH-dRCC cases displayed a greater prevalence in young patients, a higher frequency of radical nephrectomy. Seven FH-dRCC and two T2 pRCC cases received systemic therapy. The VEGF treatment was prescribed most frequently, with an objective response rate (ORR) of 22.2% and a disease control rate (DCR) of 30%. A combined therapy with VEGF and checkpoint inhibitor reported an ORR of 40% and a DCR of 100%. FH-dRCC cases showed a shortened CSS (*P* = 0.042) and DFS (*P* < 0.001). The genomic sequencing revealed 9 novel mutations.

Conclusions: Coupled with genetic detection, immunohistochemical biomarkers (FH and 2-SC) can distinguish the aggressive FH-dRCC from T2 pRCC. Future research is awaited to illuminate the association between the novel mutations and the clinical phenotypes of FH-dRCC in the disease progression.

## INTRODUCTION

As a rare subtype of renal cell carcinoma (RCC), fumarate hydratase-deficient renal cell carcinoma (FH-dRCC) is characteristic of pathologic germline/somatic mutations in the FH gene [1–3] while lesions with germline mutations are associated with hereditary leiomyomatosis and RCC (HLRCC) syndrome. The latter, an autosomal dominant disorder, usually predisposes individuals to cutaneous leiomyomas (CL), multiple uterine leiomyomas (MUL), and RCC [4–6]. Currently, FH-dRCC is recognized as a separate category in the World Health Organization (WHO) classification of RCC [7]. FH-dRCC is the preferred term for RCC with compatible morphology, negative FH immunohistochemical (IHC) staining, positive 2-SC IHC staining, and/or identification of a mutation in the FH gene in the tumor, when the clinical and family history of CL and MUL is uncertain and the genetic status is unknown [8]. Clinically, FH-dRCC is an aggressive tumor that commonly presents itself with locally advanced or metastatic disease, even in the setting of a small primary tumor, and with a high rate of malignancy progression and mortality [3, 9]. So far, National Comprehensive Cancer Network (NCCN) guidelines recommend radical nephrectomy (RN) for such patients and erlotinib plus bevacizumab for advanced patients [10]. Therefore, it is of particular significance to explore the strategies for a timely diagnosis and effective surgical intervention.

Clinically, the high aggressiveness of FH-dRCC poses a particular challenge to effective management, FH-dRCC is often misdiagnosed as type 2 papillary RCC (T2 pRCC) when only by histopathology [11–13]. After the release of 2016 WHO classification criteria, studies have documented FH deficiency in many T2 pRCC cases [14, 15]. Recent investigations into the images of papillary type II HLRCC-associated RCC and FH-dRCC have identified some distinct features that may help clinical practitioners in differentiating FH-dRCC from T2 pRCC and pathologists in IHC or even gene sequencing [16–18]. However, few studies have systematically compared the clinical features and prognostic outcomes of patients with FH-dRCC and T2 pRCC.

Therefore, the current study tackled this very issue by retrospectively analyzing the clinical data of patients with FH-dRCC and T2 pRCC in a single-center database. We found that when compared with their T2 pRCC counterparts, FH-dRCC patients reported distinct features in age, disease severity, and prognostic outcomes. Moreover, we discovered 9 novel gene mutations in the FH-dRCC cohort. These findings may be of great importance to the clinical management of FH-dRCC patients.

## RESULTS

### Clinical characteristics of FH-dRCC

We identified 16 FH-dRCC patients, and the clinical features are summarized in Table 1. The FH-dRCC group included 10 males and 6 females, aged from 20 to 75 years (median 43.5 years). 73.3% women (5/6) had a personal history of MUL, 6.3% patients (1/16) had a family history of MUL and 6.3% patients (1/16) had a family history of RCC. 31.3% patients (5/16) underwent partial nephrectomy (PN) and 68.7% patients (11/16) underwent RN.

**Table 1:** Clinical features of FH-dRCC.

**Case**	**Sex**	**Age, year**	**Clinical and family history**	**Surgery**
1	Male	52	His mother accepted surgery for multiple uterine leiomyomas	Partial nephrectomy
2	Male	28	—	Radical nephrectomy
3	Female	36	Personal history of MUL	Radical nephrectomy
4	Male	62	—	Partial nephrectomy
5	Female	44	Personal history of MUL	Radical nephrectomy
6	Female	38	Personal history of MUL	Radical nephrectomy
7	Male	62	—	Partial nephrectomy
8	Male	75	—	Partial nephrectomy
9	Female	28	Personal history of MUL	Radical nephrectomy
10	Female	32	Personal history of MUL; Her father died of RCC	Partial nephrectomy
11	Male	43	—	Radical nephrectomy
12	Male	47	—	Radical nephrectomy
13	Female	23	—	Radical nephrectomy
14	Male	74	—	Radical nephrectomy
15	Male	60	—	Radical nephrectomy
16	Male	20	—	Radical nephrectomy

Abbreviations: RCC: renal cell carcinoma; MUL: multiple uterine leiomyomas.

### Pathologic characteristics, immunohistochemical and molecular analysis of FH-dRCC

Pathologic features are summarized in Table 2. Two cases were initially recognized and diagnosed as collecting duct carcinoma (12.5%, 2/16), the remainder of cases were initially diagnosed as T2 pRCC (87.5%, 14/16). All the lesions involved were unilateral and single. The median size of primary renal tumors is 5.4 cm (range 2.8–17.0 cm). The majority of cases occurred in pT1 stage (43.8%, 7/16) and pT3 stage (43.8%, 7/16), with a minority of patients presenting in pT2 stage (1/16, 6.2%) and pT4 stage (1/16, 6.2%). 18.8% of patients (3/16) presented with regional lymph node (LN) metastasis and 12.5% patients (2/16) were metastatic at diagnosis. Among the patients, 2 cases were classified as WHO/ISUP grade 2, 8 cases as grade 3, 2 cases as grade 4, and 4 cases were unknown.

**Table 2:** Pathologic features of FH-dRCC.

**Case**	**Initial Diagnosis**	**Laterality**	**Size (cm)**	**WHO/ISUP**	**T stage**	**N Stage**	**M Stage**
1	CDC	Left	3.5	NA	T3	N0	M0
2	CDC	Right	5.8	NA	T3	N0	M0
3	T2 pRCC	Left	3.5	3	T3	N0	M0
4	T2 pRCC	Left	3.2	3	T1	N0	M0
5	T2 pRCC	Right	7.5	4	T3	N0	M0
6	T2 pRCC	Right	6.5	2	T1	N1	M0
7	T2 pRCC	Right	3	3	T1	N0	M0
8	T2 pRCC	Right	4	2	T1	N0	M0
9	T2 pRCC	Right	9.5	3	T3	N0	M0
10	T2 pRCC	Left	5	3	T1	N0	M0
11	T2 pRCC	Left	8	3	T3	N0	M0
12	T2 pRCC	Left	4	3	T3	N1	M0
13	T2 pRCC	Left	9.8	3	T2	N1	M0
14	T2 pRCC	Left	6.5	NA	T1	N0	M0
15	T2 pRCC	Left	2.8	NA	T1	N0	M1
16	T2 pRCC	Left	17.0	4	T4	N0	M1

Abbreviations: CDC: collecting duct carcinoma; T2 pRCC: type 2 papillary renal cell carcinoma; NA: not available; WHO: World Health Organization; ISUP: International Society of Urological Pathology.

**Figure 1:**
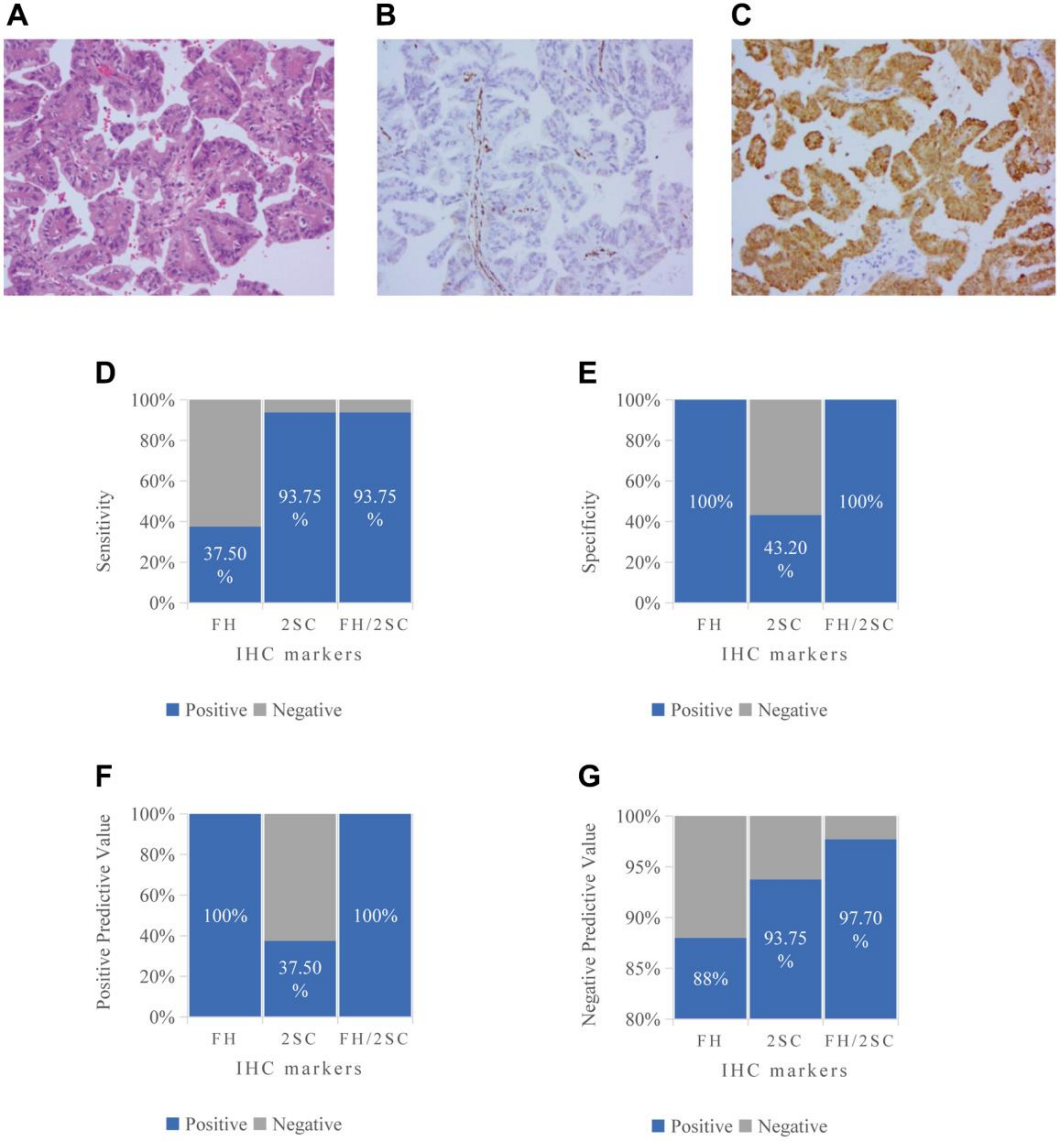
**The FH-dRCC immunohistochemistry and the preliminary results of the ability of immunohistochemistry (FH, 2-SC, FH/2-SC) to diagnose FH-dRCC.** (**A**) HE staining, 200×; (**B**) FH negative staining 200×; (**C**) 2-SC positive staining in cytoplasm/nucleus; (**D**) Sensitivity; (**E**) Specificity; (**F**) Positive Predictive Value; (**G**) Negative Predictive Value.

Immunohistochemical stains and corresponding molecular testing of 16 patients with FH-dRCC are summarized in Table 3. FH and 2-SC IHC were retrospectively performed on cases with available FFPE tissue blocks (Figure 1A–1C). Most cases showed lack of FH staining in tumor cells (62.5%, 10/16), all cases showed retained FH staining in normal kidney tissue. Most cases showed positive 2-SC staining in tumor cells (15/16, 93.8%), of which 73.3% patients (11/15) showed varying degrees of positive in cytoplasm and nuclear, and 26.7% patients (4/11) only showed different degrees of cytoplasmic positive staining. 12.5% patients (2/16) showed positive 2-SC staining in normal kidney tissue. The sensitivity, specificity, positive predictive value, and negative predictive value of FH IHC alone were determined to be 62.5%, 100%, 100%, and 88%. For 2-SC IHC staining alone, the corresponding values were 93.75%, 43.2%, 35%, and 95%. When the two markers combined, these values were found to be 93.75%, 100%, 100%, and 97.7%, respectively (Figure 1D–1G).

**Table 3:** Immunohistochemical and molecular analysis of FH-dRCC.

**Case**	**Tissue**	**FH IHC**	**2-SC IHC**	**2-SC Staining Pattern**	**FH Mutation**	**dbSNP ID**	**ACMG verdicta^a^**	**ClinVar database^b^**	**Molecular consequence**	**SIFT**	**POLUPHEN**
1	Tumor	−	+	diffuse cytoplasmic, focal nuclear	c.1108+1G>A	rs1057517734	P	P	splice donor	/	/
	Germline	+	+	diffuse cytoplasmic, dispersed nuclear	c.1108+1G>A	rs1057517734	P	P	splice donor	/	/
2	Tumor	−	+	diffuse cytoplasmic, dispersed nuclear	c.385G>A:p.E129K c.1189G>A:p.G397R	− rs863224007	US US	VUS P/LP	missense missense	T D	B D
	Germline	+	−	/	c.1189G>A:p.G397R	rs863224007	US	P/LP	missense	D	D
3	Tumor	−	+	diffuse cytoplasmic	c.556-2A>T c.845G>T:p.G282V	rs750273092 rs935002190	P US	P/LP LP	splice acceptor missense	D	D
	Germline	+	+	diffuse cytoplasmic	c.556-2A>T c.845G>T:p.G282V	rs750273092 rs935002190	US US	P/LP LP	splice acceptor missense	D	D
4	Tumor	+	+	patchy cytoplasmic	c.248G>A:p.G83D c.264G>A:p.M88I c.473G>A:p.S158N	− − rs1060500902	US US US	VUS VUS US	missense missense missense	T D D	B P D
	Germline	+	−	/	ND	/	/	/	/	/	/
5	Tumor	+	+	diffuse, nuclear and cytoplasmic	c.562A>G:p.N188D	−	US	LP	missense	D	D
	Germline	+	−	/	c.562A>G:p.N188D	−	US	LP	missense	D	D
6	Tumor	−	+	diffuse cytoplasmic, dispersed nuclear	c.1189G>A:p.G397R	rs863224007	P	P/LP	missense	D	D
	Germline	+	−	/	c.1189G>A:p.G397R	rs863224007	P	P/LP	missense	D	D
7	Tumor	+	+	focal, patchy, cytoplasmic	c.85G>A:p.G29S	−	US	VUS	missense	T	B
	Germline	+	−	/	ND	/	/	/			
8	Tumor	+	+	focal, patchy, cytoplasmic	c.267+8G>A c.272C>T:p.P91L c.302G>A:p.R101Q c.1060G>A:p.G354R	rs750447887 rs1455612736 rs75086406 −	US US LP US	LB US CIOP VUS	splice region missense missense missense	/ D T D	/ D P D
	Germline	+	-	/	ND	/	/	/	/	/	/
9	Tumor	+	+	focal nuclear and patchy cytoplasmic	c.439A>G:p.T147A	rs863223983	LP	P/LP	missense	D	P
	Germline	+	−	/	c.439A>G:p.T147A	rs863223983	LP	P/LP	missense	D	P
10	Tumor	−	+	diffuse cytoplasmic, dispersed nuclear	c.1276_1277insAGAT G:p A426fs c.1189G>A:p.G397R	− −	LP US	VUS P/LP	frame shift missense	US D	US D
	Germline	+	-	/	c.1189G>A:p.G397R	rs863224007	US	P/LP	missense	D	D
11	Tumor	+	−	/	c.1189G>A:p.G397R	rs863224007	US	P/LP	missense	D	D
	Germline	+	−	/	c.1189G>A:p.G397R	rs863224007	US	P/LP	missense	D	D
12	Tumor	−	+	diffuse cytoplasmic, dispersed nuclear	ND	/	/	/	/	/	/
	Germline	+	−	/	ND	/	/	/	/	/	/
13	Tumor	−	+	diffuse, nuclear and focal cytoplasmic	c.454A>T:p.P304fs c.911delC: p.N152Y	− −	US US	VUS VUS	missense frame shift	D US	D US
	Germline	+	−	/	ND	/	/	/			
14	Tumor	−	+	diffuse, nuclear and cytoplasmic	c.938A>T:p.E313V c.956A>T:p.D319V	− −	US US	VUS LP	missense missense	D D	D D
	Germline	+	−	/	ND	/	/	/	/	/	/
15	Tumor	−	+	diffuse, nuclear and cytoplasmic	c.289G>C:p.G97R	rs1660147877	US	US	missense	D	D
	Germline	+	−	/	ND		/	/	/		
16	Tumor	−	+	diffuse, nuclear and cytoplasmic	c.1189G>A:p.G397R	rs863224007	US	P/LP	missense	D	D
	Germline	+	−	/	c.1189G>A:p.G397R	rs863224007	US	P/LP	missense	D	D

^a^Variant pathogenicity assessment according to the American College of Human Genetics and Genomics (ACMG) guidelines (Richards et al., 2015). ^b^Pathogenicity assessments of previously reported clinical cases in the ClinVar database (https://www.ncbi.nlm.nih.gov/clinvar/); variant pathogenicity assessments that have been reviewed by expert panel are indicated with an asterisk (^*^). Abbreviations: FH: fumarate hydratase; 2-SC: 2-succino-cysteine; P: pathogenic; LP: likely pathogenic; US, uncertain significance; CIOP: conflicting interpretations of pathogenicity; VUS: variant of uncertain significance; T: tolerated; D: damaging.

Sixteen patients diagnosed with FH-dRCC underwent gene sequencing and different mutations were found in 15 cases, including 9 germline mutations and 6 somatic mutations (Table 3). No mutation was found in case 12, 9 mutations are new mutations (c.85G>A:p.G29S, c.248G>A:p.G83D, c.264G>A:p.M88I, c.385G>A:p.E129K, c.454A>T:p.P304fs, c.911delC: p.N152Y, c.938A>T:p.E313V, c.1060G>A:p.G354R, c.1276_1277insAGATG:p A426fs), which have not been reported in NCBI mutation database.

### Comparison clinical and histologic characteristics between FH-dRCC and T2 pRCC

The clinical and pathological features of FH-dRCC and T2 pRCC are summarized in Table 4. FH-dRCC and T2 pRCC showed similar distribution in sex, location, tumor size, WHO/ISUP and TNM stages. The age was significantly younger in FH-dRCC patients (45.3 ± 17.5 vs. 58.2 ± 12.7, *P* = 0.003). There were 43.8% patients under 40 years old in FH-dRCC (7/16), but only 9.1% in the T2 pRCC group (4/44). Compared with T2 pRCC, FH-dRCC patients were more likely to receive radical nephrectomy (11/16, 68.8% vs. 15/44, 34.1%, *P* = 0.021). Distant metastasis was found in 13 FH-dRCC patients, and 6 of them showed distant LN metastasis. More FH-dRCC patients developed local recurrence or metastasis after surgery (11/16, 68.8% vs. 4/44, 9.1%, *P*<0.001). A total of 3 patients with T2 pRCC had LN metastasis, and 6 had distant metastases. Both LN and distant metastases were more frequent in the FH-dRCC (*P* = 0.011 and *P* < 0.001).

**Table 4:** Comparison between FH-dRCC and T2 pRCC.

**Characteristics**	**FH-dRCC (*n* = 16)**		**T2 pRCC (*n* = 44)**		***P*-value**
Age (years), range, Mean ± SD	20–75	45.3 ± 17.5	32–82	58.2 ± 12.7	0.003^*^
Gender, *n*. %					0.558
Male	9	62.5	31	70.5	
Female	7	37.5	13	29.5	
BMI, range, Mean ± SD	17.63–27.05	21.74 ± 2.32	17.30–29.30	23.43 ± 2.70	0.03^*^
Tumor side, *n*. %					0.157
Left	10	62.5	18	40.9	
Right	6	37.5	26	59.1	
Surgery, *n*. %					0.017^*^
Partial nephrectomy	5	31.3	29	65.9	
Radical nephrectomy	11	68.7	15	34.1	
Size, median IQR	2.8–17.0	6.22 ± 3.67	0.8–17.0	4.92 ± 3.20	0.186
T stage					0.312
pT1	7	43.8	28	63.6	0.238^ʄ^
pT2	1	6.2	4	9.1	0.127^†^
pT3	7	43.8	11	25	
pT4	1	6.2	1	2.3	
N stage					0.137
N0	12	75	41	93.2	
N1	4	25	3	6.8	
M stage					0.612
M0	14	87.5	42	95.5	
M1	2	12.5	2	4.5	
WHO/ISUP					0.166
<3	2	12.5	16	36.4	
≥3	10	62.5	22	50	
Unknown	4	25	6	13.6	
Tumor FH IHC					<0.001^*^
Negative	10	62.5	0	0	
Positive	6	37.5	44	100	
Tumor 2SC IHC					0.007
Negative	1	6.2	19	43.2	
Positive	15	93.8	25	56.8	
Stage					0.027^*^
I	5	31.3	28	63.6	0.026^*‡^
II	0	0	4	9.1	0.003^*§^
III	9	56.2	9	20.5	
IV	2	12.5	3	6.8	

^*^P < 0.05; ^ʄ^pT1 vs. Others; ^†^pT1–pT2 vs. pT3–pT4; ^‡^Stage I vs. Others; ^§^Stage I–II vs. III–IV. Abbreviations: FH-dRCC: fumarate hydratase-deficient renal cell carcinoma; T2 pRCC: type 2 papillary renal cell carcinoma; BMI: Body Mass Index; WHO: World Health Organization; ISUP: International Society of Urological Pathology; FH: fumarate hydratase; 2SC: 2-succino-cysteine; IHC: immunohistochemical.

### Treatment response and survival outcomes

A total of 9 patients received systemic therapy and were evaluable for response by RECIST v1.1, including 2 metastatic T2 pRCC patients and 7 metastatic FH-dRCC patients.

One patient with T2 pRCC had metastases detected 24 months after surgery and died of the disease 59 months later after receiving chemotherapy and multiple lines of TKIs drugs. Another patient, who had bone metastatic at the time of surgery, developed lung metastases and distant LN metastases 10 months after receiving Sunitinib and died 2 months later.

The treatment response of the 7 metastatic FH-dRCC patients were summarized in Tables 5 and 6. Two patients initially diagnosed with CDC received chemotherapy but had the worst outcomes, VEGF was the most common treatment (Total, *n* = 9; Sorafenib, *n* = 1; Pazopanib, *n* = 2; Sunitinib, *n* = 3; Axitinib, *n* = 3), and showed the objective response rate (ORR) (22.2%) and DCR (55.5%). Combination therapy targeting both VEGF and checkpoint inhibitor (Total, *n* = 5; Axitinib/Toripalimab, *n* = 1; Axitinib/Tislelizumab, *n* = 1; Cabozatinib/Toripalimab, *n* = 1; Nivolumab/Ipilimumab/Cabozatinib, *n* = 1; Nivolumab/Ipilimumab, *n* = 1), and showed the ORR (40%) and DCR (100%). Bevacizumab/Erlotinib was used in 3 patients (case5, case 9, case 10). Case 10 used at third-line therapy and achieved stable disease, and case 5 and case 9 used it after resistance to multiple lines of systemic therapy and resulted in partial response (PR). This treatment achieved an ORR of 66% and a DCR of 100%. Two patients demonstrated resistance to multiple lines of systemic therapy and have changed treatment regimens, which has continued to the present.

**Table 5:** Treatment of FH-dRCC.

**Case**	**IMDC risk group (scores)**	**First line**	**Overall response/duration of treatment (mo)**	**Second line**	**Overall response/duration of treatment (mo)**	**Third line**	**Overall response/duration of treatment (mo)**
1	Intermediate (1)	Paclitaxel+Cisplatin	SD (7)	Sorafenib	PD (3)	Sunitinib	PD (3)
2	Intermediate (1)	Paclitaxel+Carboplatin	PD (NA)	Gemcitabine	PD (NA)	Doxorubicin	PD (NA)
5	Poor (3)	Sunitinib	PR (6)	Axitinib	PD (3)	Axitinib+Toripalimab	PR (16)
9	Intermediate (2)	Pazopanib	PD (9)	Axitinib+Tislelizumab	SD (4)	Cabozatinib+Toripalimab	SD (4)
10	Intermediate (2)	Nivolumab+Ipilimumab+Cabozatinib	PR (8)	Nivolumab+Ipilimumab	SD (12)	Bevacizumab+Erlotinib	SD (2)
12	Intermediate (2)	Axitinib	SD (5)	Pazopanib	SD (2)	/	/
13	Favorable (0)	Sunitinib	PR (6)	Axitinib	SD (1)	/	/

Abbreviations: PD: progressive disease; SD: stable disease; PR: partial response; NA: not available; mo: month.

**Table 6:** Prognosis of FH-dRCC.

**Case ID**	**Time at metastasis, mo**	**Metastasis at presentation**	**Therapy**	**Subsequent metastasis**	**Status**	**Follow-up, mo**
1	1 mo after nephrectomy	Bone	CT, VEGF	Peritoneal seeding, lung,	DOD	13
2	4 mo after nephrectomy	Liver	CT	Lung, distant LN, peritoneal seeding, adrenal, bone	DOD	7
3	4 mo after nephrectomy	Peritoneal seeding	Surgery	Lung	AWD	70
4	NR	—	—	—	AWOD	80
5	3 mo after nephrectomy	Lung, liver, distant LN	VEGF, ICI, VEGF +ICI	Bone, peritoneal seeding	AWD	58
6	21 mo after nephrectomy	Uterus, distant LN, pelvic, peritoneal seeding, ovary, bone	NA	liver	AWD	56
7	53 mo after nephrectomy	Peritoneal seeding	—	peritoneal seeding	AWD	53
8	NR	—	—	—	AWOD	50
9	7 mo after nephrectomy	Lung	VEGF, VEGF+ICI	distant LN, liver, peritoneal seeding, adrenal, adrenal, ovary, pelvic, uterus	AWD	47
10	22 mo after nephrectomy	Uterus, distant LN, pelvic, peritoneal seeding, ovary, liver	PD-1+CTLA-4+TKIs, PD-1+CTLA-4, VEGF + VEGF	/	AWD	44
11	NR	—	—	—	AWOD	35
12	7 mo after nephrectomy	Distant LN	VEGF	distant LN	DOD	14
13	12 mo after nephrectomy	Lung, uterus, pelvic, peritoneal seeding, bone	VEGF	/	AWD	20
14	1 mo after nephrectomy	Bone	—	bone	DOD	3
15	First onset	Lung	—	lung	AWD	4
16	First onset	Liver	—	liver	AWD	3

Abbreviations: LN: lymph node; AWOD: alive without disease; AWD: alive with disease; DOD: die of disease; DOAD: die of another disease; NA: not available; ICI: immune checkpoint inhibitor; MKI: multi-kinase inhibitor; CT: chemotherapy; mo: month.

In FH-dRCC, after a median follow-up time of 50 months (mean, 28.7 mo; range, 3 to 80 mo), 18.8% (3/16) patients showed no evidence of disease, 56.2% (9/16) patients were alive with disease, and 25% (4/16) patients died of disease. 68.8% (11/16) had recurrence and metastasis after surgery, of which 27.3% cases (3/11) underwent PN, 72.7% cases (8/11) underwent RN, 18.8% cases (3/16) had no recurrence and metastasis. Metastatic sites involved at first time included peritoneal seeding (*n* = 5), bone (*n* = 4), liver (*n* = 4), lung (*n* = 4), distant LN (*n* = 4), pelvic (*n* = 3), uterus (*n* = 3), ovary (*n* = 3). Following initial presentation, 37.5% (6/16) of patients developed subsequent metastases to distant LNs or to distant sites.

One patient underwent surgery and seven patients accepted systemic therapy when there was metastasis or progression. Among patients receiving systemic treatment, the majority of patients had an IMDC risk score of intermediate (5/7, 71.4), and a few were favorable (1/7, 14.3%) or poor (1/7, 14.3%). Of these patients, two patients died after 7 and 13 months, five patients lived with disease from 20 to 58 months with systemic therapy and one patient lived with disease in 70 months after resection of the metastatic cancers. Of the other patients, two patients died after 3 and 14 months, respectively, without timely adjuvant therapy. Three lived without recurrence from 35 to 80 months without any adjuvant therapy, two patients were unevaluable due to short follow-up time even with distant metastasis at first onset.

During the 37-month median follow-up, 68.8% (11/16) and 9.1% (4/44), patients with FH-dRCC and T2 pRCC underwent disease recurrence and metastasis. A total of 10 patients died from various causes, 4 in FH-dRCC and 6 in T2 pRCC. Of the T2 PRCC patients, 1 died of malnutrition, 1 died of bone malignant tumor, and 4 died of metastatic T2 pRCC. These 4 patients were diagnosed with metastasis RCC before surgery, 2 of them were treated with TKIs and died at 12 and 83 months after diagnosis.

**Figure 2:**
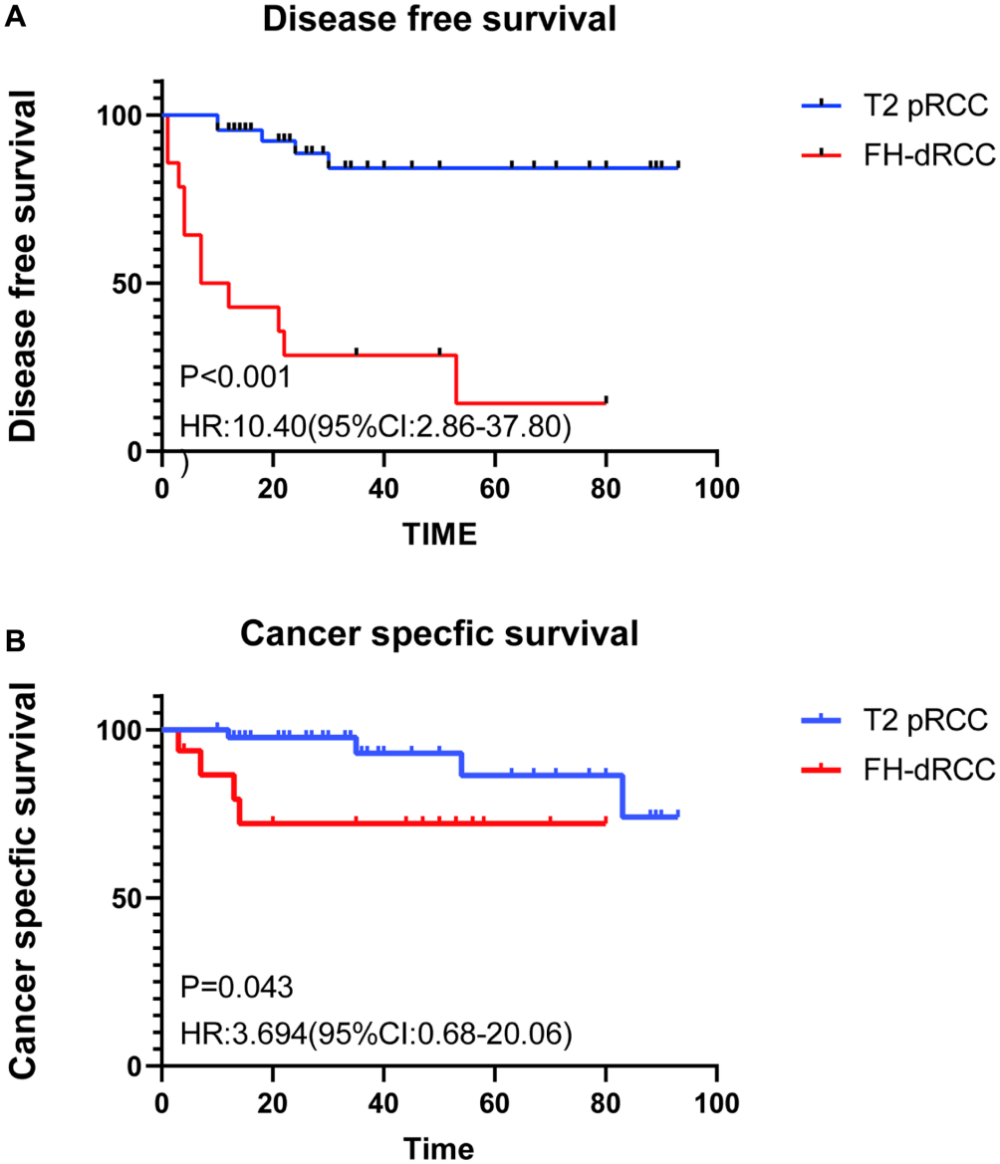
**Comparison of survival between FH-dRCC patients and T2 pRCC.** (**A**) DFS of FH-dRCC and T2 pRCC; (**B**) CSS of FH-dRCC and T2 pRCC).

Figure 2 exhibits the Kaplan–Meier plots for disease-free survival (DFS) and cancer-specific survival (CSS) estimates stratified by FH-dRCC versus T2 pRCC. FH-dRCC patients had a shorter DFS (*P* < 0.001, Figure 2A). The median DFS of FH-dRCC and T2 pRCC was 9.5 month and not reached. Similarly, FH-dRCC patients were at significantly higher risk for cancer-specific mortality than patients with T2 pRCC (*P* = 0.042, Figure 2B). The median CSS of FH-dRCC and T2 pRCC was not reached. The 1-year, 3-year, 5-year cancer-specific survival rates were 87.5%, 75%, and 75% for FH-dRCC and 97.7%, 95.5%, and 93.2% for T2 pRCC.

## DISCUSSION

FH-dRCC is a rare and recently described entity tumor with variable clinical and pathologic features and closely related to the RCC found in HLRCC syndrome [19–21]. At present, observations suggest that the clinical behavior of FH-dRCC and HLRCC-associated RCC is similar. [2, 11, 14]. All of FH-dRCC cases in our cohort had no known family history of HLRCC and due to the lack of definitive genetic or clinical information at their initial presentations, we use the term “FH-dRCC” to include both HLRCC-associated RCC and disseminated cases of FH-dRCC.

This study analyzed the baseline characteristics, immunohistochemical features, gene mutation and prognosis of 16 FH-dRCC patients and compared with those of 44 patients with T2 pRCC. Many of our findings support the conclusions described in previous studies [14, 18], including that FH-dRCC tend to present at a younger age than T2 pRCC (median age of 43.5 years), behave aggressively (50% of cases presented at tumor stage pT3a or higher) and more common in males. Lymph node and distant metastases were more frequent in FH-dRCC, as shown in the present study, metastases are commonly observed in the LNs, liver, lungs, bone and peritoneal seeding, and portend a poor prognosis. To date, a total of 14 FH-dRCC younger than 20 years old have been reported, but the disease is not limited in younger patients [22]. In this study, 9 of 16 patients (56.3%) were older than 40 years, and in a previous study [3], 62.5% patients were older than 40 years old. In addition, MUL were frequently observed in FH-dRCC, whereas CL were not observed. Similarly, CL are rare in patients with HLRCC in Japan and China. The phenotype of HLRCC may differ between Asians and Caucasians [1, 23, 24]. HLRCC only presented as single CL is usually disregarded and misdiagnosed. The participants in this study were RCC patients. Patients with personal and family history of MUL all had germline mutations, while patients with somatic mutations had no history of MUL, CL and RCC.

Although pathological features play a pivotal role in the diagnosis of FH-dRCC, it may be confused with T2 pRCC when only through microscopic examination, FH and 2-SC IHC is a useful tool. Studies have shown that FH-/2-SC+ has optimal sensitivity and specificity for FH-dRCC, the sensitivity even reaching 100% [14, 25]. In this study, we performed FH and 2-SC IHC in all patients and sensitivity and specificity were also 93.75% and 100% when FH and 2-SC were used together. Most of FH-dRCC (68.8%) showed FH staining deficiency and the vast majority (93.8%, 15/16) showed 2-SC staining positivity, of which 73.3% showed cytoplasmic and nuclear staining to varying degrees and 26.7% showed only cytoplasmic positive staining, one of the patients confirmed by gene test showed FH+/2-SC- with a meaningful mutation (c.1189G>A:p.G397R), similar cases have never been reported in previous articles. While in 56.8% of T2 pRCC patients was cytoplasmic staining only in 2-SC immunoreactivity but no FH mutation was detected. While Chen’s study showed that all confirmed cases showed diffuse and strong nuclear and cytoplasmic staining, a small subset of unclassified and T2 pRCC exhibited immunoreactivity to 2-SC, but did not harbor FH germline or somatic alterations. That is, FH gene mutation may also be detected in other RCC when 2-SC IHC was cytoplasmic staining only. Therefore, the diagnosis of 2-SC IHC staining pattern in FH-dRCC needs to be more cautious. Other IHC stains also are useful in conjunction with FH/2-SC IHC in distinguishing FH-deficient RCC from its morphologic mimics, such as CK7, p63, and GATA3 in differentiating FH-deficient RCC from CDC [3, 26]. Zhang et al. found that AKR1B10 is a new sensitive and specific marker for FH-dRCC, which in conjunction with 2-SC and FH can help in the diagnosis of the disease and reduce the rate of leakage and misdiagnosis [27].

As of November 2023, the ClinVar database includes 268 pathogenic and 147 likely pathogenic single gene variants within the FH gene [28]. This data still being updated. In our study, 21 different FH gene mutations were detected. Two are frame shift mutations, 16 are missense mutations, 3 are splicing positions, 9 are recent discoveries. Of the 9 newly discovered mutations, 3 are damaging, 3 are benign, and 3 are still uncertain. Germline mutations were found in both patients with a personal and family history, as well as in other three patients without any history. Immunohistochemistry of tumor specimens from 1 patient suggested FH-/2-SC-, but no mutation was found, and we suspected large fragment deletion.

As found in our study, the prognosis of FH-dRCC is worse than T2 pRCC. The proportion of patients with distant metastasis was higher in our study (81.3% vs. 13.6%) than in the study by Yang, while that of LNs metastasis was lower (37.5% vs. 6.8%) [18]. Since FH-dRCC is significantly more invasive than T2 pRCC, RN and lymph node dissection are recommended.

A study showed the median survival for metastatic disease was 18 months [9]. In another report [3], a small number of (19%, 5/26) patients with FH-dRCC showed no evidence of disease, a portion of (31%, 8/26) patients were alive with disease, and most patients (50%, 13/26) were died of disease after a median follow-up of 16 months (range, 1 to 118 mo) in 26 patients. However, in this study, 18.8% of patients had no postoperative recurrence or metastasis, which may be related to low DNA methylation, most patients survived. This may be related to a relatively low genome-wide DNA methylation [29]. Xu’s study [30] found that ICI/TKI combination therapy was associated with more favorable outcomes compared to other first-line therapies, including Bevacizumab/Erlotinib combination therapy and TKI monotherapy, suggesting a combination of immunotherapy and TKI could be promising in advanced FH-dRCC. Lucia’s study [31] found that ORR for treatments were 50% for cabozantinib, 43% for sunitinib, 63% for other antiangiogenics, and 30% for Bevacizumab/Erlotinib, whereas ORR was 0% for mTOR inhibitors and 18% for ICBs. In our study, only a subset (43.75%, 7/16) received systemic treatment and all developed resistance, disease progression, even death. But ICI/TKI combination therapy was associated with more favorable outcomes compared to other first-line therapies, suggesting a combination of immunotherapy and targeted therapy could be promising in advanced FH-dRCC. We also tried Axitinib/Toripalimab, Lenvatinib/Tislelizumab, Cabozantinib/Toripalimab, Lenvatinib/Everolimu and other treatment protocols, failed but no death. Interestingly, 18.8% patients are alive without disease and 1 patient with lung metastases discovered shortly after resection of the metastases survived without receiving systemic therapy and did not show disease progression, which may be related to the relatively good prognosis morphology. Compared with targeted therapy alone, immune-based combination medication can improve the ORR and DCR of patients. Dong’s [32] study also found that in patients with germline mutations, the ORR and DCR of immune-based treatment were higher. This is because there is abundant CD8+T cell infiltration in fumarate hydratase-deficient renal cell carcinoma, and the depletion of T cells is related to poor immune efficacy in FH-dRCC patients. Current anti-PD-1 /PD-L1 antibodies cannot reverse the depletion of CD8+T cells in such patients. In addition, Liang [33] et al. observed abnormalities in several negative immunomodulators, which may be related to immune escape. However, there have been no studies on the correlation between FH-dRCC mutation sites and treatment programs. In our study, case 5 (c.562A>G:p.N188D) and case 9 (c.439A>G:p.T147A) showed no response to systemic treatment such as TKIs, PD-1 or PD-1/TKIs and developed resistance in a short time. Patients with mutation (c.1189G>A:p.G397R) was the most common (31.3%). Although it was reported to be damaging in previous studies, 60% of patients didn’t show strong aggressiveness, however patients with mutation (c.385G>A:p.E129K and c.1189G>A:p.G397R) died after receiving chemotherapy. Patients with mutation (c.1276_1277insAGATG:p A426fs and c.1189G>A:p.G397R) experienced PR soon after receiving PD-1/CTLA-4 and TKIs, with more than 50% reduction in focal size. Therefore, we hypothesized that patients with c.1189G>A:p.G397R and c.1276_1277insAGATG:p A426fs mutations would benefit more. The newly discovered mutation in case 13 (c.454A>T:p.P304fs, c.911delC: p.N152Y), despite multiple metastases, was PR after treatment with TKIs and is now stable. Given the limited number of cases, it is not possible to draw definitive conclusions regarding the relationship between gene variations and drug sensitivity or prognostic assessment in patients. However, the diverse range of gene variations identified in this study will serve as a foundation for building a comprehensive understanding of FH-dRCC through future molecular typing studies. These findings contribute to the initial stages of research in this field, facilitating further exploration and characterization of FH-dRCC.

Our study also has some limitations. First, the limited sample size and short follow-up period may lead to inaccurate statistical results and prognostic judgments. However, this is still the first time that the largest sample of FH-dRCC has been considered for reproductive/physiological mutations and compared with T2 pRCC in terms of prognosis. Second, in some cases, we could not obtain confirmation of the presence of FH mutations in uterine smooth muscle tumors, nor could we obtain a complete family history regarding specific HLRCC features.

## MATERIALS AND METHODS

### Patients

**Figure 3:**
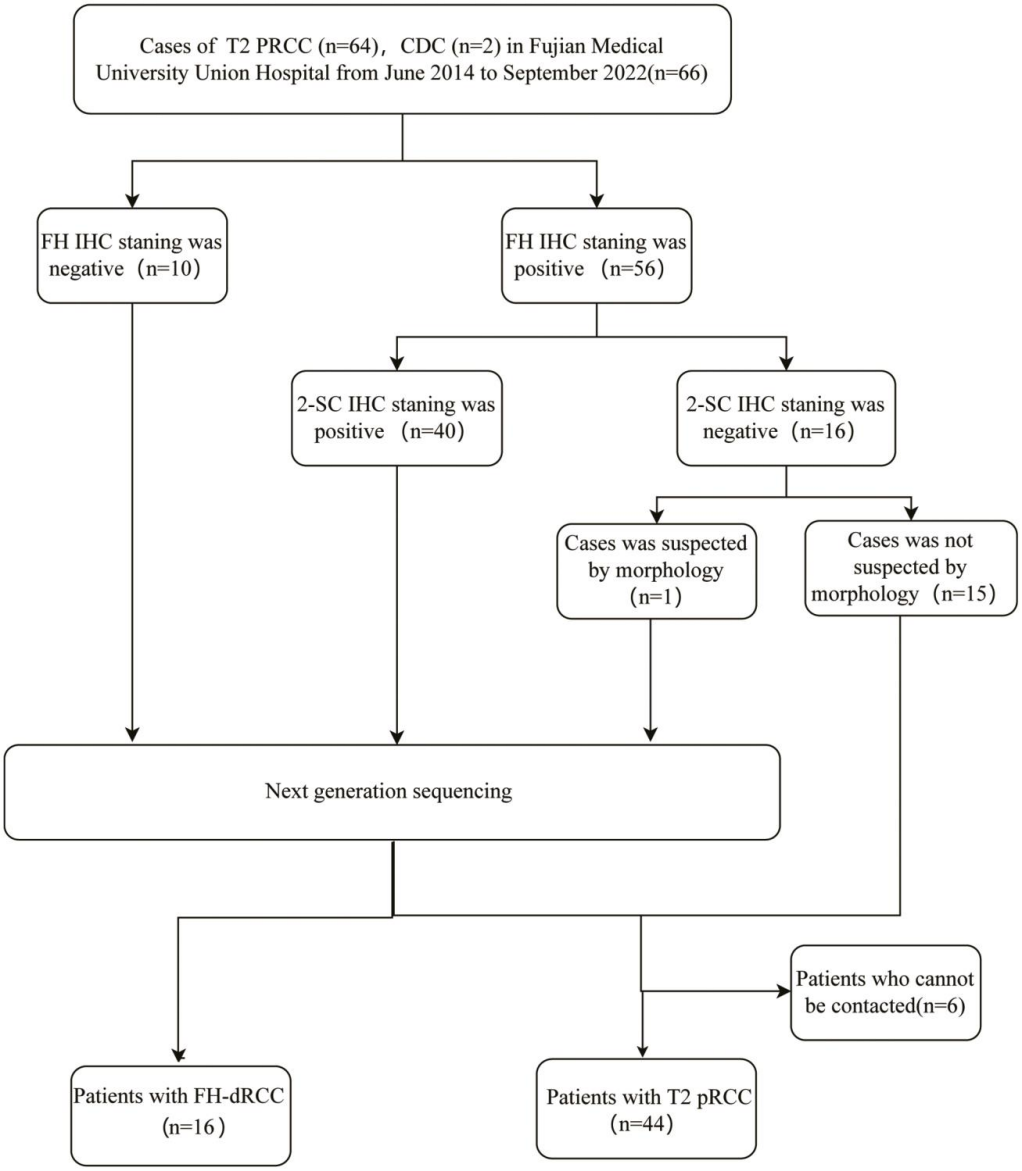
Flowchart of patient selection.

Ethical approval for this study (Ethical Committee NO.2019KJCX037) was provided by the Ethical Review Committee of Fujian Medical University Union Hospital on 28 November 2019 and all eligible participants provided written informed consent. It recruited from June 2014 to September 2022, 16 FH-dRCC patients with pathological IHC FH-confirmed RCC (defined as FH- and/or 2-SC+) or FH mutation and 44 pathologically-diagnosed T2 pRCC patients. Clinical data were collected, including patients’ age, sex, tumor size, surgical data, pathological results, staging, treatment, follow-up time, outcome, and family history of CL or MUL or RCC within secondary consanguinity. The flowchart of patient selection was shown in Figure 3.

### Histologic features

Each tumor was diagnosed by two experienced expert pathologists. The tumor specimens were respectively stained and analyzed using FH antibody reagents and 2-SC antibody reagents, which were ready-to-use kits purchased from Fuzhou Maixin Biotechnology Development Co., Ltd. In the non-tumor renal parenchyma, when an internal positive control in inflammatory cells or interstitial cells, or a FH-positive staining in cytoplasm was present, tumor cells with FH negative staining were considered negative. When adjacent non-tumor cells showed a 2-SC-negative staining (internal negative control), tumor cells with 2-SC positive staining in cytoplasm/nucleus were considered positive. Histological types of tumors were categorized according to the 2016 classification of the WHO, and tumors were graded according to the WHO/International Society of Urological Pathology (ISUP) grading system.

### Mutational analyses

ANNOVAR (http://annovar.openbioinformatics.org/en/latest/) was referred to for all the released mutations with the latest group database, function, database, and known disease information such as comparative analyses. The following SNV/InDel sites were consulted for evaluation of variation frequency, functional characteristics, conservation, and pathogenicity (A. Population databases: dbSNP, 1000Genomes, ESP6500, ExAC03, ExAC03_EAS, gnomAD, Hrcr1, Kaviar, WBBC, Genesky Exon DB_Freq, Genesky Genome DB_Freq; B. Gene annotation: MalaCards, GO BP/CC/MF, KEGG pathway, Protein interaction; C. Disease related databases: GWAS, COSMIC, OMIM, HGMD, ClinVar, MGI, MalaCards, HPO; D. Conservation and protein hazard prediction: SIFT, POLYPhen V2, MutationTaster, MetaSVM, BayesDel addAF, ClinPred, Cadd Dann, VEST, REVEL).

### Follow up

Patients were followed up with regular postoperative visits with radiological surveillance imaging of the chest, abdomen and pelvis was performed at various time intervals. The last information on file was considered as the final follow-up time. The primary endpoint (PE) of the study was cancer-specific survival (CSS), which was defined as the length from the date of surgery to death from renal cell carcinoma, and the secondary endpoint (SE) was the disease-free survival (DFS), which was defined as the interval from the date of nephrectomy until the diagnosis of tumor recurrence, metastasis or last follow-up. The main therapeutic effects of systemic treatment were ORR and DCR.

### Statistical analysis

All statistical analyses were performed using IBM SPSS version 26.0 (IBM Corporation, Armonk, NY, USA). Continuous and categorical variables were expressed as mean ± SD and percentage, respectively, and compared between groups by unpaired Student’s *t*-test or chi-square test. PE and SE were assessed by Kaplan-Meier estimation and log-rank test. Statistical significance was defined as a two-tailed *P*-value < 0.05.

### Availability of data and materials

The datasets used and analyzed during the current study are available from the corresponding author on reasonable request.

## CONCLUSION

In conclusion, the clinicopathological parameters and oncological prognosis of FH-dRCC differ from those of T2 pRCC, and we confirm many findings of previous studies, as well as some current cases, which have uncommon features. FH-dRCC has a younger age of onset and more rapid disease progression, and immunohistochemistry of FH and 2-SC can distinguish well between the two, considering that not all cases of FH-dRCC cases show FH staining deficiency and diffuse 2-SC staining in immunohistochemical assays, FH gene mutation analysis should still be considered in patients with suspicious clinical or pathological features. FH-dRCC is aggressive, has a poor prognosis, and there is no uniform treatment protocol. ICI combined with TKIs has brought benefits to the treatment of this disease, but there are significant inter-individual differences in efficacy, the reasons for which still need to be actively explored.

## References

[1] Pan X, Zhang M, Yao J, Zeng H, Nie L, Gong J, Chen X, Xu M, Zhou Q, Chen N. Fumaratehydratase-deficient renal cell carcinoma: a clinicopathological and molecular study of 13 cases. J Clin Pathol. 2019; 72:748–54. 10.1136/jclinpath-2019-205924 31262952

[2] Kuroda N, Tsutsui M, Iguchi M, Nobuoka E, Uehara T, Sonobe Y, Morinaga Y, Shibuya S, Oda W, Yanai H, Kawada C, Karashima T, Yamasaki I, et al. Fumarate hydratase-deficient renal cell carcinoma: A clinicopathological study of seven cases including hereditary and sporadic forms. Ann Diagn Pathol. 2020; 49:151599. 10.1016/j.anndiagpath.2020.151599 32977234

[3] Lau HD, Chan E, Fan AC, Kunder CA, Williamson SR, Zhou M, Idrees MT, Maclean FM, Gill AJ, Kao CS. A Clinicopathologic and Molecular Analysis of Fumarate Hydratase-deficient Renal Cell Carcinoma in 32 Patients. Am J Surg Pathol. 2020; 44:98–110. 10.1097/PAS.0000000000001372 31524643

[4] Gupta S, Erickson LA. Fumarate Hydratase-Deficient Renal Cell Carcinoma. Mayo Clin Proc. 2020; 95:619–21. 10.1016/j.mayocp.2020.01.026 32138894

[5] Reed WB, Walker R, Horowitz R. Cutaneous leiomyomata with uterine leiomyomata. Acta Derm Venereol. 1973; 53:409–16. 4127477

[6] Launonen V, Vierimaa O, Kiuru M, Isola J, Roth S, Pukkala E, Sistonen P, Herva R, Aaltonen LA. Inherited susceptibility to uterine leiomyomas and renal cell cancer. Proc Natl Acad Sci U S A. 2001; 98:3387–92. 10.1073/pnas.051633798 11248088 PMC30663

[7] Moch H, Amin MB, Berney DM, Compérat EM, Gill AJ, Hartmann A, Menon S, Raspollini MR, Rubin MA, Srigley JR, Hoon Tan P, Tickoo SK, Tsuzuki T, et al. The 2022 World Health Organization Classification of Tumours of the Urinary System and Male Genital Organs-Part A: Renal, Penile, and Testicular Tumours. Eur Urol. 2022; 82:458–68. 10.1016/j.eururo.2022.06.016 35853783

[8] Wyvekens N, Valtcheva N, Mischo A, Helmchen B, Hermanns T, Choschzick M, Hötker AM, Rauch A, Mühleisen B, Akhoundova D, Weber A, Moch H, Rupp NJ. Novel morphological and genetic features of fumarate hydratase deficient renal cell carcinoma in HLRCC syndrome patients with a tailored therapeutic approach. Genes Chromosomes Cancer. 2020; 59:611–9. 10.1002/gcc.22878 32537760

[9] Muller M, Ferlicot S, Guillaud-Bataille M, Le Teuff G, Genestie C, Deveaux S, Slama A, Poulalhon N, Escudier B, Albiges L, Soufir N, Avril MF, Gardie B, et al. Reassessing the clinical spectrum associated with hereditary leiomyomatosis and renal cell carcinoma syndrome in French FH mutation carriers. Clin Genet. 2017; 92:606–15. 10.1111/cge.13014 28300276

[10] NCCN Clinical Practice Guidelines in Oncology (NCCN Guidelines®) Kidney Cancer Version 1.2024. https://www.nccn.org/patients.

[11] Skala SL, Dhanasekaran SM, Mehra R. Hereditary Leiomyomatosis and Renal Cell Carcinoma Syndrome (HLRCC): A Contemporary Review and Practical Discussion of the Differential Diagnosis for HLRCC-Associated Renal Cell Carcinoma. Arch Pathol Lab Med. 2018; 142:1202–15. 10.5858/arpa.2018-0216-RA 30281371

[12] Merino MJ, Torres-Cabala C, Pinto P, Linehan WM. The morphologic spectrum of kidney tumors in hereditary leiomyomatosis and renal cell carcinoma (HLRCC) syndrome. Am J Surg Pathol. 2007; 31:1578–85. 10.1097/PAS.0b013e31804375b8 17895761

[13] Yu YF, He SM, Wu YC, Xiong SW, Shen Q, Li YY, Yang F, He Q, Li XS. [Clinicopathological features and prognosis of fumarate hydratase deficient renal cell carcinoma]. Beijing Da Xue Xue Bao Yi Xue Ban. 2021; 53:640–6. 10.19723/j.issn.1671-167X.2021.04.003 34393221 PMC8365061

[14] Trpkov K, Hes O, Agaimy A, Bonert M, Martinek P, Magi-Galluzzi C, Kristiansen G, Lüders C, Nesi G, Compérat E, Sibony M, Berney DM, Mehra R, et al. Fumarate Hydratase-deficient Renal Cell Carcinoma Is Strongly Correlated With Fumarate Hydratase Mutation and Hereditary Leiomyomatosis and Renal Cell Carcinoma Syndrome. Am J Surg Pathol. 2016; 40:865–75. 10.1097/PAS.0000000000000617 26900816

[15] Taylor AS, Skala SL. Tumors masquerading as type 2 papillary renal cell carcinoma: pathologists' ever-expanding differential diagnosis for a heterogeneous group of entities. Urol Oncol. 2022; 40:499–511. 10.1016/j.urolonc.2021.04.043 34116938

[16] Paschall AK, Nikpanah M, Farhadi F, Jones EC, Wakim PG, Dwyer AJ, Gautam R, Merino MJ, Srinivasan R, Linehan WM, Malayeri AA. Hereditary leiomyomatosis and renal cell carcinoma (HLRCC) syndrome: Spectrum of imaging findings. Clin Imaging. 2020; 68:14–9. 10.1016/j.clinimag.2020.06.010 32562921 PMC8916163

[17] Nikolovski I, Carlo MI, Chen YB, Vargas HA. Imaging features of fumarate hydratase-deficient renal cell carcinomas: a retrospective study. Cancer Imaging. 2021; 21:24. 10.1186/s40644-021-00392-9 33608050 PMC7893914

[18] Yang L, Li XM, Hu YJ, Zhang MN, Yao J, Song B. Multidetector CT Characteristics of Fumarate Hydratase-Deficient Renal Cell Carcinoma and Papillary Type II Renal Cell Carcinoma. Korean J Radiol. 2021; 22:1996–2005. 10.3348/kjr.2021.0212 34668351 PMC8628156

[19] Trpkov K, Hes O. New and emerging renal entities: a perspective post-WHO 2016 classification. Histopathology. 2019; 74:31–59. 10.1111/his.13727 30565301

[20] Ohe C, Smith SC, Sirohi D, Divatia M, de Peralta-Venturina M, Paner GP, Agaimy A, Amin MB, Argani P, Chen YB, Cheng L, Colecchia M, Compérat E, et al. Reappraisal of Morphologic Differences Between Renal Medullary Carcinoma, Collecting Duct Carcinoma, and Fumarate Hydratase-deficient Renal Cell Carcinoma. Am J Surg Pathol. 2018; 42:279–92. 10.1097/PAS.0000000000001000 29309300 PMC8015937

[21] Peng YC, Chen YB. Recognizing Hereditary Renal Cancers Through the Microscope: A Pathology Update. Surg Pathol Clin. 2018; 11:725–37. 10.1016/j.path.2018.07.010 30447838

[22] Hol JA, Jongmans MCJ, Littooij AS, de Krijger RR, Kuiper RP, van Harssel JJT, Mensenkamp A, Simons M, Tytgat GAM, van den Heuvel-Eibrink MM, van Grotel M. Renal cell carcinoma in young FH mutation carriers: case series and review of the literature. Fam Cancer. 2020; 19:55–63. 10.1007/s10689-019-00155-3 31792767 PMC7026215

[23] Noguchi G, Furuya M, Okubo Y, Nagashima Y, Kato I, Matsumoto K, Tanaka R, Hisasue SI, Yao M, Kishida T. Hereditary leiomyomatosis and renal cell cancer without cutaneous manifestations in two Japanese siblings. Int J Urol. 2018; 25:832–5. 10.1111/iju.13760 30058172

[24] Kuwada M, Chihara Y, Lou Y, Torimoto K, Kagebayashi Y, Tamura K, Shuin T, Fujimoto K, Kuniyasu H, Samma S. Novel missense mutation in the FH gene in familial renal cell cancer patients lacking cutaneous leiomyomas. BMC Res Notes. 2014; 7:203. 10.1186/1756-0500-7-203 24684806 PMC3978052

[25] Muller M, Guillaud-Bataille M, Salleron J, Genestie C, Deveaux S, Slama A, de Paillerets BB, Richard S, Benusiglio PR, Ferlicot S. Pattern multiplicity and fumarate hydratase (FH)/S-(2-succino)-cysteine (2SC) staining but not eosinophilic nucleoli with perinucleolar halos differentiate hereditary leiomyomatosis and renal cell carcinoma-associated renal cell carcinomas from kidney tumors without FH gene alteration. Mod Pathol. 2018; 31:974–83. 10.1038/s41379-018-0017-7 29410489

[26] Liu Y, Dong Y, Gu Y, Xu H, Fan Y, Li X, Dong L, Zhou L, Yang X, Wang C. GATA3 aids in distinguishing fumarate hydratase-deficient renal cell carcinoma from papillary renal cell carcinoma. Ann Diagn Pathol. 2022; 60:152007. 10.1016/j.anndiagpath.2022.152007 35841867

[27] Zheng L, Zhang X, Pan X, Huang Z, Zhang M, Xian J, Wei Y, Nie L, Zhang M, Gong J, Chen X, Zhou Q, Zeng H, Chen N. AKR1B10 Is a New Sensitive and Specific Marker for Fumarate Hydratase-Deficient Renal Cell Carcinoma. Mod Pathol. 2023; 36:100303. 10.1016/j.modpat.2023.100303 37580017

[28] NCBI. Fh[Gene] - CliVar - NCBI. n.d. Accessed November 1, 2022. https://www.ncbi.nlm.nih.gov/clinvar/?gr=0&term=FH%5Bgene%5D&redir=gene.

[29] Sun G, Zhang X, Liang J, Pan X, Zhu S, Liu Z, Armstrong CM, Chen J, Lin W, Liao B, Lin T, Huang R, Zhang M, et al. Integrated Molecular Characterization of Fumarate Hydratase-deficient Renal Cell Carcinoma. Clin Cancer Res. 2021; 27:1734–43. 10.1158/1078-0432.CCR-20-3788 33414138

[30] Xu Y, Kong W, Cao M, Wang J, Wang Z, Zheng L, Wu X, Cheng R, He W, Yang B, Dong B, Pan J, Chen Y, et al. Genomic Profiling and Response to Immune Checkpoint Inhibition plus Tyrosine Kinase Inhibition in FH-Deficient Renal Cell Carcinoma. Eur Urol. 2023; 83:163–72. 10.1016/j.eururo.2022.05.029 35715365

[31] Carril-Ajuria L, Colomba E, Cerbone L, Romero-Ferreiro C, Crouzet L, Laguerre B, Thibault C, Vicier C, de Velasco G, Fléchon A, Saldana C, Benusiglio PR, Bressac-de Paillerets B, et al. Response to systemic therapy in fumarate hydratase-deficient renal cell carcinoma. Eur J Cancer. 2021; 151:106–14. 10.1016/j.ejca.2021.04.009 33975058

[32] Dong P, Zhang X, Peng Y, Zhang Y, Liu R, Li Y, Pan Q, Wei W, Guo S, Zhang Z, Han H, Zhou F, Liu Y, He L. Genomic Characteristics and Single-Cell Profiles After Immunotherapy in Fumarate Hydratase-Deficient Renal Cell Carcinoma. Clin Cancer Res. 2022; 28:4807–19. 10.1158/1078-0432.CCR-22-1279 36074152

[33] Liang J, Sun G, Pan X, Zhang M, Shen P, Zhu S, Zhao J, Zheng L, Zhao J, Chen Y, Yin X, Chen J, Hu X, et al. Genomic and transcriptomic features between primary and paired metastatic fumarate hydratase-deficient renal cell carcinoma. Genome Med. 2023; 15:31. 10.1186/s13073-023-01182-7 37131267 PMC10152735

